# Machine learning application in personalised lung cancer recurrence and survivability prediction

**DOI:** 10.1016/j.csbj.2022.03.035

**Published:** 2022-04-04

**Authors:** Yang Yang, Li Xu, Liangdong Sun, Peng Zhang, Suzanne S. Farid

**Affiliations:** a Department of Biochemical Engineering, University College London, Gower Street, London WC1E 6BT, UK; b Department of Thoracic Surgery, Shanghai Pulmonary Hospital, Tongji University School of Medicine, Shanghai 200043, China

**Keywords:** Machine learning, Decision tree, Lung cancer, Personalized diagnosis and prognosis, ANNs, artificial neural networks, ANOVA, analysis of variance, AUC, the area under the ROC curve, CART, classification and regression tree, CNV, copy number variation, DTs, decision trees, FFNN, Feedforward neural networks, LS-SVM, least-squares support vector machine, LUAD, lung adenocarcinoma, LUSC, lung squamous cell carcinoma, NSCLC, non-small cell lung cancer, ML, machine learning, ROC, receiver operating characteristic, SVMs, support vector machines, TCGA, The Cancer Genome Atlas, TNM, a common cancer staging system while T, N and M refers to tumour, node and metastasis

## Abstract

Machine learning is an important artificial intelligence technique that is widely applied in cancer diagnosis and detection. More recently, with the rise of personalised and precision medicine, there is a growing trend towards machine learning applications for prognosis prediction. However, to date, building reliable prediction models of cancer outcomes in everyday clinical practice is still a hurdle. In this work, we integrate genomic, clinical and demographic data of lung adenocarcinoma (LUAD) and squamous cell carcinoma (LUSC) patients from The Cancer Genome Atlas (TCGA) and introduce copy number variation (CNV) and mutation information of 15 selected genes to generate predictive models for recurrence and survivability. We compare the accuracy and benefits of three well-established machine learning algorithms: decision tree methods, neural networks and support vector machines. Although the accuracy of predictive models using the decision tree method has no significant advantage, the tree models reveal the most important predictors among genomic information (e.g. KRAS, EGFR, TP53), clinical status (e.g. TNM stage and radiotherapy) and demographics (e.g. age and gender) and how they influence the prediction of recurrence and survivability for both early stage LUAD and LUSC. The machine learning models have the potential to help clinicians to make personalised decisions on aspects such as follow-up timeline and to assist with personalised planning of future social care needs.

## Graphical abstract

**Figure:**
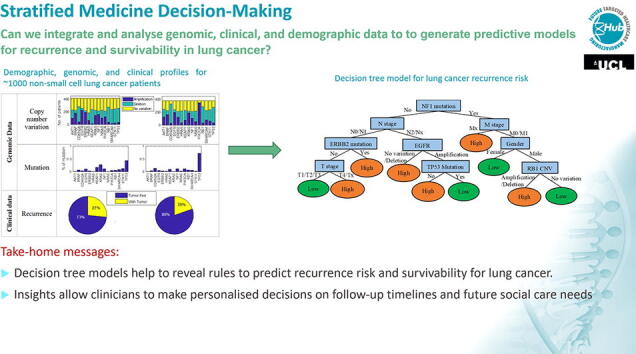

## 1 Introduction

Lung cancer is the most commonly diagnosed cancer globally and the leading cause of cancer death in both sexes combined with an estimated 1.6 million deaths in 2018 [1]. Approximately 85% of patients have a group of histological subtypes collectively known as non-small cell lung cancer (NSCLC), of which lung adenocarcinoma (LUAD) and lung squamous cell carcinoma (LUSC) are the most common subtypes [2]. Lobar resection is the standard curative modality for early-stage (Stage I and II) and selected Stage III NSCLC [3]. Although the treatment of NSCLC has made great progress in the past few decades, the five-year survival rate has not improved significantly due to the initial diagnosis at a late stage. Moreover, the recurrence after surgery usually occurs very rapidly: 50–90% occur two years after surgery, and 90–95% of patients occur within five years [4]. Currently, the popularity of computed tomography has significantly increased the rate of early screening for lung cancer [5]. However, there is still a lack of a systematic and objective approach for better diagnosis and treatment of NSCLC. The present study aims to integrate genomic, clinical, diagnostic and demographic data to generate a full picture of patients in order to develop a risk prediction model for the overall survival and recurrence status for NSCLC.

The identification of individuals’ overall survival and relapse requires an accurate and robust predictive model. Machine learning (ML) techniques can discover and identify patterns and relationships between various variables from complex datasets so as to predict effectively future outcomes of many cancers. ML has been widely applied to cancer prognosis and prediction [6], [7], [8], [9], [10], [11], [12], [13], [14]. It has been reported that ML methods can be used to substantially (15–25%) improve the accuracy of predicting cancer susceptibility, recurrence and mortality [6]. In NSCLC, most previous predictive models for lung cancer have been developed based on risk factors such as tobacco smoking history, family history of lung cancer and occupational exposures [15], [16], [17]. However, these conventional risk factors generally do not provide enough information to make robust predictions or prognoses [6]. With the rapid development of genomic, proteomic and imaging technologies, more specific molecular scale information about the tumour and the patient have been discovered as powerful indicators of cancer prognosis and prediction [18], [19]. In addition to well-known biomarkers of KRAS and EGFR, new genomic biomarkers, such as somatic mutations in ALK, ERBB2, TP53 have been demonstrated to be associated with lung cancer risk, response or prognosis [20], [21], [22]. As proteomic biomarkers, 17 circulating inflammatory proteins have demonstrated clinical utility in lung cancer prognosis [23], [24], [25], [26]. Most recently, many researchers have analysed the quantitative features from radiological images and correlated the radiomic biomarkers with lung cancer prognosis and mutation status [27], [28], [29], [30].

All the emerging biomarkers act as new pieces of the lung cancer puzzle. Ideally, for accurate and robust prediction of an individual’s cancer prognosis, all pieces need to fit into the puzzle to generate the full picture of the patient. This means integrating carefully all histological, clinical, demographic, genomic, proteomic, metabolic and radiomic information to come up with a reasonable prognosis. Chen et al. [31] attempted to assess the survival prediction of NSCLC patients through the use of artificial neural networks (ANNs) with 10 selected genes expression as well as clinical and demographic data (sex, age, T stage and N stage). Hanai et al. [32] applied ANNs to construct a prognostic model for 125 NSCLC patients with 12 clinico-pathological variables (age, sex, smoking index, tumor size, p factor, pT, pN, stage, histology) and 5 immunohistochemical variables (p27 percentage, p27 intensity, p53, cyclin D1, retinoblastoma). Hsia et al [33] investigated the survival time in advanced lung cancer patients using ANNs from the genetic polymorphism of the p21 and p53 genes in conjunction with patients' general data (gender, age, disease type and period of lung cancer, chemical diagnosis, treatment type of chemical diagnosis, smoking habit). Marchevsky et al. [34] predicted the survival of Stage I and II NSCLC patients using clinical-pathological (age, sex, cell type, stage, tumour grade, smoking history) and immunohistochemical variables (c-erbB-3, bcl-2, Glut1, Glut3, retinoblastoma gene and p53).

The objective of our present study was to develop a risk prediction model using ML methods to predict overall survival and recurrence status for NSCLC using The Cancer Genome Atlas (TCGA) cohorts. The distinctive features of the work include integrating genomic, clinical and demographic data and introducing both copy number variation (CNV) and mutation information of a broader set of genes (15) to predict overall survival and recurrence status for both LUAD and LUSC patients. The 15 selected genes (TP53, STK11, KRAS, KEAP1, EGFR, SMARCA4, CDKN2A, BRAF, RB1, PIK3CA, NF1, ERBB2, HRAS, NRAS, AKT1) for constructing the predictive model were chosen based on reports of their significance for NSCLC [35], [36]. Mutations in a number of these genes may contribute to NSCLC and represent potential therapeutic targets for these tumours. For example, targeted antibody therapies for lung cancer with mutant EGFR oncogenes include necitumumab (Portrazza, Eli Lilly and Co.), cetuximab (Erbitux, Eli Lilly and Co.) and amivantamab-vmjw (Rybrevant, Janssen Biotech).

## 2 Data and methods

### 2.1 TCGA data description

**Fig. 1:**
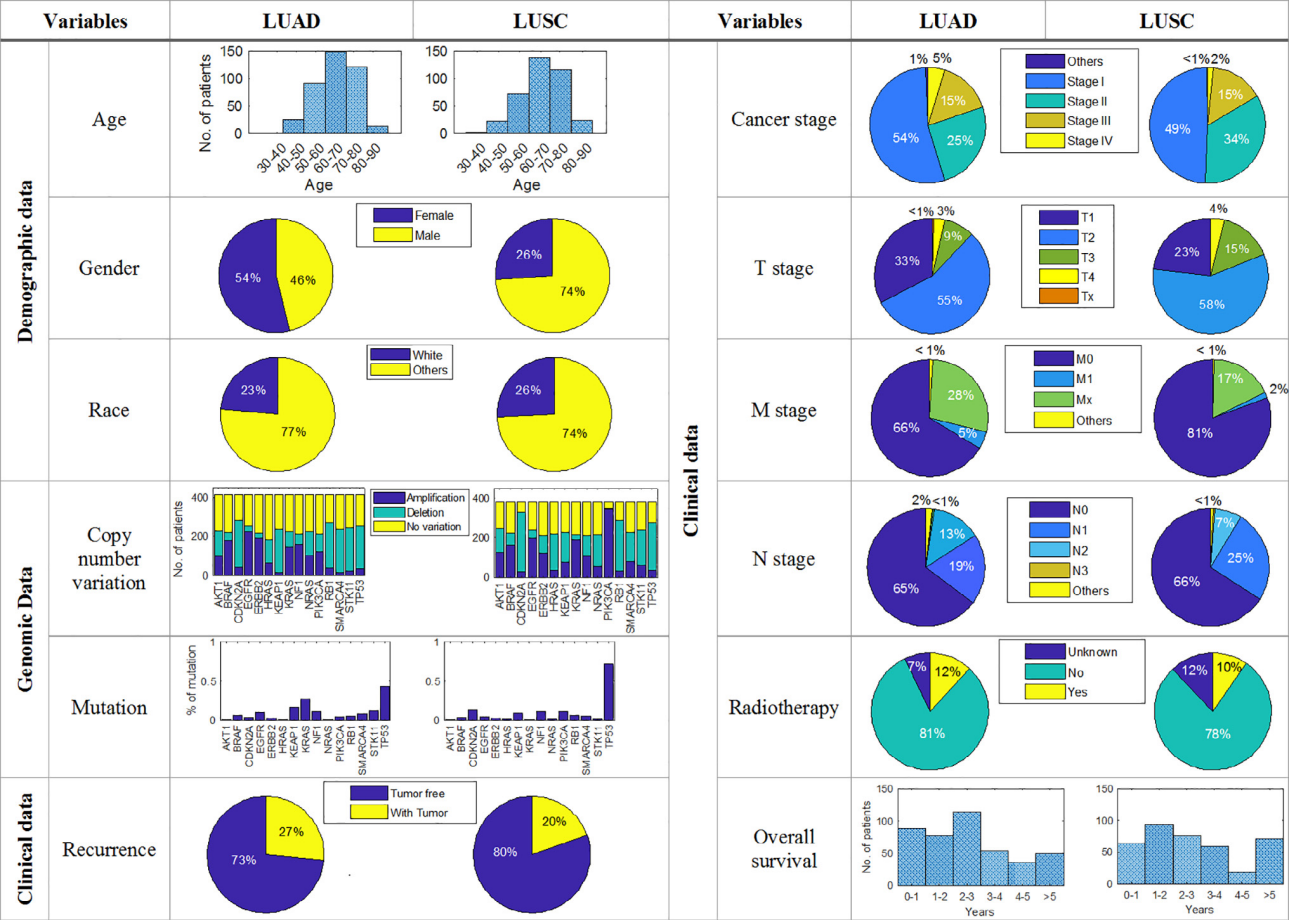
Demographic, genomic and clinical profiles of TCGA dataset for non-small cell lung cancer (418 LUAD and 382 LUSC patients).

TCGA is the largest public pan-cancer biology database, which is available from the TCGA Data Portal at https://tcga-data.nci.nih.gov/tcga/. TCGA database includes genomic, transcriptomic, and epigenetic data for 33 human cancer types represented with more than 11,000 individual samples. In this work, we focus on NSCLC with two major subtypes: LUAD and LUSC. Altogether, we collected 511 representative samples of LUAD and 487 LUSC for which genomic, clinical and demographic data are available for both subtypes. Demographic data includes Age, Gender and Race. Clinical data includes Cancer Stage, TNM Stage, History of Prior Cancer Diagnosis, Overall Survival and Recurrence Status. Genomic data includes mutation and CNV information of 15 genes: TP53, STK11, KRAS, KEAP1, EGFR, SMARCA4, CDKN2A, BRAF, RB1, PIK3CA, NF1, ERBB2, HRAS, NRAS and AKT1. Among the NSCLC data, only Age and Overall Survival are two numerical variables. According to the average value, the Age and the Overall Survival variables were transformed into two categories, namely < or ≥ 65 years and < or ≥ 3 years respectively. For the clinical cancer stage variables (Cancer Stage and TNM Stage), we only consider the major stages from I to IV but not the subdivision stages like IA, IB. Fig. 1 summarize the TCGA data used in this work.

### 2.2 Analysis of variance (ANOVA)

ANOVA is a procedure for determining whether variation in the response variable arises within or among different population groups. In this work, one-way analysis of variance is used to determine whether there are any statistically significant differences between the means of NSCLC factors. The level of statistical significance is expressed as the p-value, which is the probability of observing the sample results given that the null hypothesis is true. Usually, a p-value threshold of 0.05 can be considered as statistically significant.

### 2.3 Decision trees (DTs)

DTs are important, well-established machine learning techniques, which have been used for a wide range of applications, especially for classification problems [37], [38].

In this work, a popular decision tree algorithm, CART (classification and regression tree), was applied to construct binary trees [39]. The Gini index was used as the splitting rule for CART. If costs of misclassification are not specified, the Gini index is defined as:

(1) $$g\left(t\right)=\;\underset{j\neq i}{\sum }p\left(j|t\right)p\left(i|t\right)$$

If costs of misclassification are specified, then the Gini index is defined as:

(2) $$g\left(t\right)=\;\underset{j\neq i}{\sum }C\left(i|j\right)p\left(j|t\right)p\left(i|t\right)$$

where the sum extends over all *k* categories. $\text{p}\left(\text{j}/\text{t}\right)$ is the probability of category *j* at the node *t* and $\text{C}\left(\text{i}/\text{j}\right)$ is the probability of misclassifying a category *j* case as category *i*.

(3) $$I_{G}\left(f\right)=\sum_{i=1}^{m}f_{i}\left(1-f_{i}\right)=\sum_{i=1}^{m}f_{i}-\sum_{i=1}^{m}f_{i}^{2}=1-\sum_{i=1}^{m}f_{i}^{2}$$

The tree structure has been optimized based on the best accuracy found using 10-fold cross-validation in MATLAB (R2017).

### 2.4 Artificial neural networks (ANNs)

ANNs are a set of algorithms, to simulate the functioning of a human brain, that are designed to recognize patterns, which result in data-driven models that can interpret effectively patterns in multivariate data from non-linear systems [40].

In this study, a common neural network algorithm, the feedforward neural network (FFNN) [41], [42], was applied to construct a model with one hidden layer of 20 neurons using MATLAB (R2017). The maximum number of epochs for training was set to 1000. To prevent the trained network model from over-training, the training procedure stopped if the validation performance degraded for 10 consecutive epochs. The optimal trained network with the best validation performance was selected. The training function used in this work was the Levenberg-Marquardt algorithm which was designed to solve non-linear least squares problems [43]. The Levenberg-Marquardt algorithm uses the Jacobian matrix in the following Newton-like update:

(4) $$x_{k+1}=x_{k}-{\left[J^{T}J+\mu I\right]}^{-1}J^{T}e$$

where *J* is the Jacobian matrix that contains first derivatives of the network errors with respect to the weights and biases, and ***e*** is a vector of network errors. If the scalar µ is zero, this is just Newton's method, using the approximate Hessian matrix. If µ is large, this becomes gradient descent with a small step size. Thus, µ is decreased after each successful and is increased only when a tentative step would increase the performance function.

### 2.5 Support vector machines (SVMs)

SVMs are supervised learning methods in machine learning algorithms for classification and regression analysis [44].

Least-squares support vector machine (LS-SVM) [45] was used to construct non-linear classification models in this work using MATLAB (R2017). In this work, the optimal regression line (y=w·φ(x)+b) was found by minimizing the object function in Equation (5) while *w* and *b* are the regression weight coefficients and the bias terms of the final model.

(5) $$Q=\frac{1}{2}w^{T}w+\frac{1}{2}\overset{\;\;\;N}{\underset{\;\;\;i=1}{\Gamma \sum }}e_{i}^{2}$$

*e*_*i*_ is the error tolerance of the model. In this work, two parameters, г and σ^2^, required by implementation of LS-SVM were set as 5 and 2 accordingly.

A summary of the pros and cons of the three machine learning approaches compared in this analysis is provided in Table 1.

**Table 1:** Comparison of machine learning methods.

**Methods**	**Pros**	**Cons**
**Decision tree**	• Easy to understand • Efficient training • Can be used for classification or regression • Order of training instances has no effect on training • Pruning can deal with the problem of overfitting	• Classes must be mutually exclusive • Final decision tree dependent upon order of attribute selection • Errors in training set can result in overly complex decision trees • Missing values for an attribute make it unclear about which branch to take when that attribute is tested
**Neural network**	• Can be used for classification or regression • Able to represent Boolean functions • Tolerant of noisy inputs • Instances can be classified by more than one output	• Difficult to understand structure of algorithm • Too many attributes can result in overfitting • Optimal network structure can only be determined by experimentation
**Support vector machine**	• Models nonlinear class boundaries • Overfitting is unlikely to occur • Computational complexity reduced to quadratic optimization problem • Easy to control complexity of decision rule and frequency of error	• Training is slow compared to decision trees • Difficult to determine optimal parameters when training data is not linearly separable • Difficult to understand structure of algorithm

### 2.6 *K*-fold cross-validation

*K*-fold cross-validation is a widely used technique for assessing the robustness of a model [46]. In *k*-fold cross-validation, the original sample is randomly partitioned into *k* equal size subsets. Of the *k* subsets, a single subset is retained as the validation data for testing the model, and the remaining *k*-1 subsets are used as training data. The cross-validation process is then repeated *k* times (the folds) and the *k* results from the folds can be averaged to produce a single estimation. The advantage of this method over repeated random sub-sampling is that all observations are used for both training and validation and each observation is used for validation exactly once. In this work, 10-fold cross-validation was applied for all three machine learning methods for estimating the prediction error.

## 3 Results and discussion

This section presents insights from machine learning methods on identification and prediction of key factors for recurrence and survivability of LUSC and LUAD. Variance analysis is used to reveal the factors with significant influence on recurrence and survivability. These findings are compared with previous studies for corroboration. Three common ML methods (decision trees, neural networks and support vector machines) are applied for building predictive models and their performance is compared in terms of their ability to accurately predict recurrence and survivability of LUSC and LUAD.

### 3.1 Analysis of variance on recurrence risk and survivability for NSCLC

**Fig. 2:**
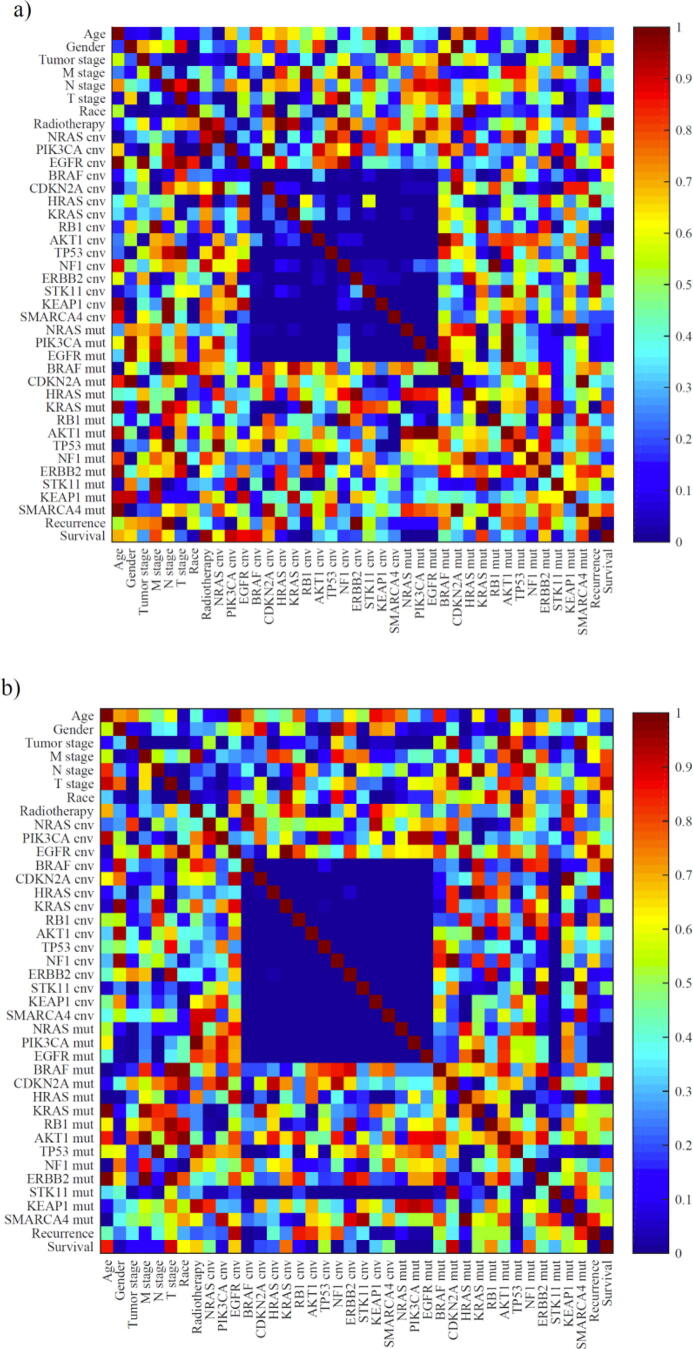
The p-value matrix for all ANOVA tests two subtypes of NSCLC for (a) LUAD and (b) LUSC. cnv = copy number variation, mut = mutation.

The matrix of p-values for all one-way ANOVA tests for two subtypes of NSCLC, (a) LUAD and (b) LUSC, are shown in Fig. 2. The p-value matrix reveals the statistically significant impact of copy number variation types (amplification or deletion) between the 15 genes investigated in the work. The signalling pathways involved in the development of lung cancer can explain this observation. The key gene mutations in these pathways are correlated with each other. Therefore, the expression of each key transcription factor may cause a series of downstream factors and cross-protein changes [47], [48], [49], [50]. A similar result [51] reveals that the miR-3151 gene (miRNA gene family) is driven by BRAF-independent mechanisms while the TP53 gene could act as a downstream effector of miR-3151. This finding provided evidence for a causal link between BRAF mutations and TP53.

Statistically significant demographic, clinical and genomic factors for recurrence and overall survival of NSCLC are indicated in Table 2. From the demographic data, race was identified as a significant factor for both LUSC recurrence and overall survivability, with patients of white race having a lower recurrence rate but also a lower survival rate compared to patients of other races. The influence of race on incidence and survival of NSCLC has been attributed to the diversity in inherited genetic variations and an accumulation of somatic genetic events [52], [53]. LUSC overall survivability was also found to be influenced by gender with female patients tending to have higher survival rates than male. This observation is reinforced by other works [54], [55]. The mechanism leading to the difference between genders is still unknown, but endocrine factors are believed to play an important role [56], [57]. It is worth noting that no significant difference was found in the different age groups (<65 and ≥ 65) for either recurrence rate or survival rate for LUSC and LUAD, although age is a well-known risk factor for development and progression of cancer [58].

**Table 2:** Significant demographic, clinical and genomic factors for recurrence and overall survival of non-small cell lung cancer.

Significant factors			LUSC						LUAD					
			Recurrence			Overall Survival			Recurrence			Overall Survival		
			Yes (%)	P	Sig.	≥3y (%)	P	Sig.	Yes (%)	P	Sig.	≥3y (%)	P	Sig.
Demographic	Age	<65	20.5	0.830		27.6	0.450		26.9	0.642		12.8	0.640	
		≥65	21.5			31.4			29.1			18.2		
	Gender	Female	16.5	0.199		41.0	0.029	*	29.6	0.486		14.9	0.580	
		Male	23.0			27.5			26.3			16.7		
	Race	Other	30.4	0.034	*	53.8	8E-05	***	32.9	0.331		20.0	0.222	
		White	18.7			26.1			27.0			14.8		
Clinical	Cancer stage	I	15.9	0.001	**	37.2	0.140		13.2	4E-15	***	5.8	0.137	
		II	20.2			22.9			38.8			25.0		
		III	40.9			36.4			46.2			28.1		
		IV	33.3			0.00			78.9			72.7		
	M stage	M0	20.4	0.563		35.9	3E-04	***	27.2	0.523		13.1	0.790	
		M1	33.3			0.00			78.9			72.7		
		Mx	23.3			9.10			21.7			24.3		
	T stage	T1	13.2	0.025	*	34.2	0.027	*	17.3	1E-05	***	10.0	0.278	
		T2	22.9			35.7			31.0			16.0		
		T3	21.2			11.9			41.4			32.0		
		T4	50.0			16.7			54.5			20.0		
	N stage	N0	16.7	0.008	**	29.6	0.117		18.8	8E-06	***	10.1	0.788	
		N1	25.9			30.3			45.3			26.9		
		N2	47.8			57.1			46.7			29.6		
		N3	----			----			50.0			28.1		
		Nx	0.00			50.0			----			72.7		
	Radio-Therapy	No	19.6	0.044	*	31.5	0.845		23.3	1E-07	***	13.3	0.485	
		Yes	34.3			29.6			59.2			37.9		
Genomic	EGFR CNV	A	18.1	0.143		40.0	0.012	*	30.0	0.496		16.1	0.199	
		D	21.6			9.10			21.4			8.3		
		N	25.2			26.0			26.8			16.8		
	KRAS CNV	A	23.8	0.231		30.4	0.398		30.5	0.657		16.0	0.026	*
		D	20.8			10.5			24.7			13.8		
		N	18.1			35.5			27.9			16.4		
	NF1 mutation	No	18.6	0.001	**	29.2	0.024	*	27.2	0.297		14.7	0.124	
		Yes	41.7			50.0			34.9			23.5		
	ERBB2 mutation	No	20.1	0.004	**	31.0	0.418		28.5	0.321		16.1	0.267	
		Yes	62.5			50.0			12.5			0.0		
	STK11 mutation	No	21.1	0.851		31.3	0.939		26.2	0.017	*	14.8	0.080	
		Yes	25.0			33.3			43.9			25.0		
	TP53 mutation	No	22.2	0.774		19.2	0.007	**	23.1	0.013	*	13.6	0.982	
		Yes	20.8			36.0			34.8			19.1		
	KEAP1 mutation	No	20.6	0.440		30.7	0.570		26.1	0.043	*	15.5	0.160	
		Yes	26.7			37.5			39.0			17.1		
	SMARCA4 mutation	No	20.6	0.313		30.5	0.14		26.7	0.037	*	15.4	0.594	
		Yes	31.2			38.5			44.8			21.1		

Note: P refers to the p-value of ANOVA analysis that indicates the statistical significance of each factor. Sig. refers to the significance level of p-value: 0.01 < p < 0.05 (*), 0.001 < p < 0.01(**), p < 0.001(***).

Regarding clinical predictors, cancer stage, N stage and radiotherapy were found to be significant factors for recurrence for both LUAD and LUSC. T stage was identified as a significant factor for recurrence for both LUAD and LUSC but as a significant factor for survival for LUSC only. The analysis indicated that M stage was a significant factor for survival for LUSC only. As is well known, the current cancer staging and TNM staging system for lung cancer are both essential for predicting prognosis and selecting appropriate treatment; it is derived by the International Association for the Study of Lung Cancer from a database of 94,708 patients from 46 sites across 19 countries [59], [60]. Usually, patients with a high cancer stage or TNM stage have a poor prognosis and high recurrence risk [60]. This work found that patients with adjuvant radiotherapy had a significantly higher recurrence rate for both LUAD and LUSC. This observation can be explained by the fact that radiotherapy is usually given to the patients in the advanced or terminal stage before or after surgery due to the high relapse rate [61].

Table 2 also highlights the significant genomic factors. The analysis indicates that the EGFR and KRAS copy number variation can have a significant impact on survival rate for LUSC and LUAD respectively while the mutation status of NF1, ERBB2, STK11, TP53, KEAP1 and SMARCA4 can be significant factors. EGFR mutations have been used as the basis for targeted therapies such as EGFR tyrosine kinase inhibitors (EGFR-TKIs) and antibodies. The EGFR pathway is one of the recently discovered pathways that can promote lung cancer. Mutations in the EGFR gene can lead to an increase in the degree of malignancy of lung cancer. There is a significant association between sensitivity to EGFR TKIs and the types of EGFR mutations [62]. Globally, KRAS mutant tumours are the most common potential overlapping molecular subtypes in non-small cell lung cancer [63]. From a clinical point of view, KRAS-mutant lung cancer is usually associated with a worse overall survival rate than KRAS wild-type tumours, especially in advanced cancers [64], [65]. However, other studies in the early stage [66] or late group [67] were inconsistent in confirming this poor survival; therefore, the prognostic significance of KRAS mutation status in lung cancer remains a controversial topic. However, recent biologic findings in KRAS, coupled with the advent of immunotherapy, may lead to the development of effective therapeutic strategies and optimal therapeutic stratification of the KRAS-mutant NSCLC in the near future [68], [69]. With regard to other gene mutations highlighted in this work, such as NF1, ERBB2, STK11, TP53, KEAP1 and SMARCA4, they also play important roles in various pathways associated with the metastasis or overall survival [70], [71], [72]. However, there is still a lack of effective inhibitors to block their expression.

### 3.2 Early stage (Stage I & II) NSCLC recurrence risk prediction

In the NSCLC dataset, there are 276 records of early stage LUSC and 295 records of early stage LUAD with recurrence information. In order to build up a predictive model with machine learning methods, some pre-processing work of NSCLC data is necessary. First, patient records with null values or missing values have been removed from the training dataset. Second, a set of class labels have been given to the records of training dataset since all the machine learning methods applied in this work are supervised learning methods. According to the recurrence status, each patient record was classified into one of two groups: High risk and Low risk. Table 3 summarises the number of records under each classification in the training dataset of the recurrence risk for LUSC and LUAD.

**Table 3:** Summary of machine learning training datasets used for recurrence risk for LUAD and LUSC.

Class labels	Description	No. of records	
		LUSC	LUAD
High risk	tumour recurrence after initial resection treatment	49	64
Low risk	no tumour recurrence after initial resection treatment	227	231

**Fig. 3:**
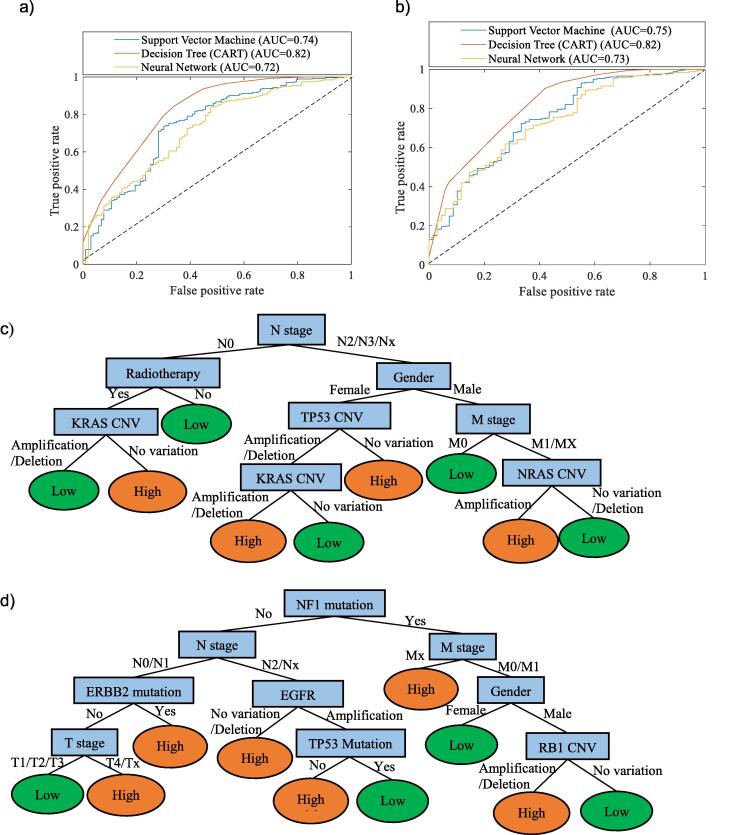
Receiver operating characteristic (ROC) curve of performance comparison of NSCLC recurrence risk models using different machine learning algorithms for (a) LUAD and (b) LUSC recurrence risk prediction. Decision tree (CART) model for (c) LUAD and (d) LUSC recurrence risk prediction.

To compare the performance of different machine learning methods (CART, FFNNs and LS-SVM), for prediction of early stage NSCLC recurrence risk, the receiver operating characteristic (ROC) curve for each method was generated for LUAD and LUSC respectively (Fig. 3(a) and (b)). The ROC curve is a common method to demonstrate the diagnostic ability of a binary classifier system by plotting the true positive rate against the false positive rate at various threshold settings. The threshold refers to a boundary between the classes of a classifier system [73]. The diagonal line from the bottom left to the top right in a ROC curve represents random guessing. The point in the upper left corner, (0, 1), represents the best possible prediction method with 100% sensitivity and 100% selectivity. To compare the average performance of different classifiers, it is common to calculate the area under the ROC curve (AUC) as an average performance indicator. AUC is a portion of the area of the unit square therefore its value is between 0 (worst performance) and 1 (perfect performance). From the ROC curves, it shows that the decision tree models have the best performance in recurrence prediction for both LUAD and LUSC with AUC values of 0.82 in both cases. The LS-SVM and the FFNN models have similar performance (AUC = 0.72–0.75).

The CART models in Fig. 3(c) and (d) revealed the key factors on recurrence risk for LUAD and LUSC accordingly. Furthermore, from top to bottom along the branch to each leaf node of the tree, the “if-then” rules can be generated to describe and predict whether a patient has a high or low risk of recurrence. For example, in Fig. 3(c), the left branch of the tree indicates that if a LUAD patient is in N0 stage, with adjuvant radiation therapy treatment experience and has CNV in KRAS (either in deletion or amplification), then the recurrence risk is low. From the predicted models, N stage and M stage are two important determinants of recurrence risk for both cancer subtypes, which reinforce the ANOVA analysis results in Section 4.1. As discussed before, the TNM staging indicates the level of disease progression and the malignant potential of the primary lung cancer. However, the TNM staging system may reach the limit of its usefulness in recurrence risk prediction since even patients with disease at the same stage exhibit wide variations in their incidence of recurrence after curative resection [74]. The decision tree models indicated that gender in demographics as well as N stage and M stage in clinical status are the common recurrence risk for both LUAD and LUSC. Adjuvant radiation therapy is more effective in LUAD rather than LUSC. For LUSC recurrence risk, the CNV types in KRAS, TP53 and NRAS play important roles. For LUAD recurrence risk, mutation in NF1, ERBB2 and TP53 and CNV types in EGFR and RB1 are important. The predictive models in this work have the potential to help clinicians to accurately predict the cases in which disease is likely to recur and to make personalised clinical approach schedule and follow-up timeline.

### 3.3 Early stage (Stage I & II) NSCLC survivability prediction

In the NSCLC dataset, there are 242 records of early stage LUSC and 249 records of early stage LUAD with survivability information. The pre-processing work of survivability training dataset is the same as recurrence risk prediction mentioned before. According to the overall survival length after initial resection treatment, each patient record was classified into one of two groups: Good and Poor. The summary of training dataset of the survivability is shown in Table 4.

**Table 4:** Summary of machine learning training datasets used for survivability for LUAD and LUSC.

Class labels	Description	No. of records	
		LUSC	LUAD
Good	overall survival **≥** 3 years after initial resection treatment	75	68
Poor	overall survival < 3 years after initial resection treatment	167	181

**Fig. 4:**
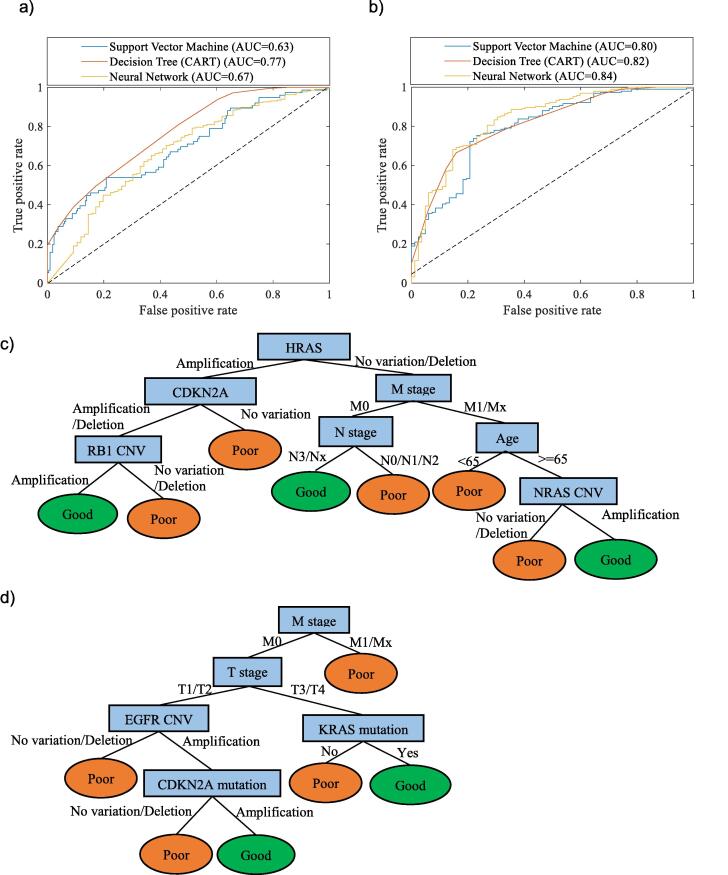
Receiver operating characteristic (ROC) curve of performance comparison of NSCLC survivability models using different machine learning algorithms for (a) LUAD and (b) LUSC survivability prediction. Decision tree (CART) model for (c) LUAD and (d) LUSC survivability prediction.

The ROC curves for the three machine learning methods are shown in Fig. 4 (a) and (b) for LUAD and LUSC respectively. From the ROC curves, it shows that the decision tree model has the best performance in survivability prediction for LUAD with the AUC values as 0.767 while the neural network is slightly better than decision tree in LUSC survivability prediction with the AUC values as 0.837 and 0.815 respectively.

The decision tree models in Fig. 4(c) and (d) revealed the key factors for survivability for LUAD and LUSC accordingly. Similar to the results in Fig. 4, from top to bottom along the branch to each leaf node of the tree, the “if-then” rules can be generated to describe and predict whether a patient is in good or poor survivability. Although the TNM stage plays an important role in survivability for both cancer subtypes, the decision tree models in this work revealed that M stage in clinical status is the common impactor of survivability of early stage NSCLC. Age and N stage are more important for LUAD rather than LUSC while T stage is more important for LUSC rather than LUAD. For genomics information, the CNV types in HRAS, CDKN2A, RB1 and NRAS play important roles in LUAD survivability while the mutation status in CDKN2A and KRAS as well as CNV types in EGFR are important in LUSC survivability. The potential of the tree models in this work offers support for clinicians to predict the individual survivability and to assist with personalised planning of future social and care needs.

## 4 Conclusion

The major contribution of this work is the construction of a more complete portrait of NSCLC patients by integrating genomic, clinical and demographic data when building predictive models using machine-learning methods. By comparing these three methods, CART tree models demonstrate good predictive performance and advantages in understandable tree-like graphs that can generate the rules to predict recurrence and survivability for LUSC and LUAD. The key factors and if-then rules revealed by the tree models can provide clinicians with a better understanding of recurrence risk and overall survivability of early stage NSCLC. The results of this work also have the potential to help clinicians to make personalised decisions on tailored treatment and follow-up plans.

## Declaration of Competing Interest

The authors declare that they have no known competing financial interests or personal relationships that could have appeared to influence the work reported in this paper.

## References

[1] Bray F. (2018). Global cancer statistics 2018: GLOBOCAN estimates of incidence and mortality worldwide for 36 cancers in 185 countries. CA Cancer J Clin.

[2] Molina J.R. (2008). Non-small cell lung cancer: epidemiology, risk factors, treatment, and survivorship. Mayo Clin Proc.

[3] Hirsch F.R. (2016). Lung cancer: current therapies and new targeted treatments. Lancet.

[4] Alberts W.M. (2007). Follow up and surveillance of the patient with lung cancer: what do you do after surgery?. Respirology.

[5] Goldstraw P. (2007). The IASLC Lung Cancer Staging Project: proposals for the revision of the TNM stage groupings in the forthcoming (seventh) edition of the TNM Classification of malignant tumours. J Thorac Oncol.

[6] Cruz J.A., Wishart D.S. (2006). Applications of machine learning in cancer prediction and prognosis. Cancer Inform.

[7] Cicchetti D.V. (1992). Neural networks and diagnosis in the clinical laboratory: state of the art. Clin Chem.

[8] Uramoto H., Tanaka F. (2012). Prediction of recurrence after complete resection in patients with NSCLC. Anticancer Res.

[9] Exarchos K.P., Goletsis Y., Fotiadis D.I. (2012). Multiparametric decision support system for the prediction of oral cancer reoccurrence. IEEE Trans Inf Technol Biomed.

[10] Kononenko I. (2001). Machine learning for medical diagnosis: history, state of the art and perspective. Artif Intell Med.

[11] Park K. (2013). Robust predictive model for evaluating breast cancer survivability. Eng Appl Artif Intell.

[12] Sun Y.J. (2007). Improved breast cancer prognosis through the combination of clinical and genetic markers. Bioinformatics.

[13] Levitsky A. (2019). Early symptoms and sensations as predictors of lung cancer: a machine learning multivariate model. Sci Rep.

[14] Lai Y.-H. (2020). Overall survival prediction of non-small cell lung cancer by integrating microarray and clinical data with deep learning. Sci Rep.

[15] Marcus M.W. (2015). LLPi: Liverpool lung project risk prediction model for lung cancer incidence. Cancer Prev Res (Phila).

[16] Park S. (2013). Individualized risk prediction model for lung cancer in Korean men. PLoS ONE.

[17] Bach P.B. (2003). Variations in lung cancer risk among smokers. JNCI-J Nat Cancer Inst.

[18] Vargas A.J., Harris C.C. (2016). Biomarker development in the precision medicine era: lung cancer as a case study. Nat Rev Cancer.

[19] Qian Z. (2015). Nuclear factor, erythroid 2-like 2-associated molecular signature predicts lung cancer survival. Sci Rep.

[20] Butkiewicz D. (2001). Genetic polymorphisms in DNA repair genes and risk of lung cancer. Carcinogenesis.

[21] Li Y.F. (2010). Genetic variants and risk of lung cancer in never smokers: a genome-wide association study. Lancet Oncol.

[22] Mechanic L.E. (2007). Common genetic variation in TP53 is associated with lung cancer risk and prognosis in African Americans and somatic mutations in lung tumors. Cancer Epidemiol Biomark Prev.

[23] Alifano M. (2011). Preresection serum C-reactive protein measurement and survival among patients with resectable non-small cell lung cancer. J Thorac Cardiovasc Surg.

[24] Enewold L. (2009). Serum concentrations of cytokines and lung cancer survival in African Americans and Caucasians. Cancer Epidemiol Biomark Prev.

[25] Hong S. (2012). Elevated Serum C-Reactive Protein as a Prognostic Marker in Small Cell Lung Cancer. Yonsei Med J.

[26] Zhou T., Wang T., Garcia J.G.N. (2014). Expression of nicotinamide phosphoribosyltransferase-influenced genes predicts recurrence-free survival in lung and breast cancers. Sci Rep.

[27] Thawani R. (2018). Radiomics and radiogenomics in lung cancer: A review for the clinician. Lung Cancer.

[28] Yu K.H. (2016). Predicting non-small cell lung cancer prognosis by fully automated microscopic pathology image features. Nat Commun.

[29] Peeken J.C. (2018). Radiomics in radiooncology - Challenging the medical physicist. Phy Med-Eur J Med Phys.

[30] Arimura H. (2019). Radiomics with artificial intelligence for precision medicine in radiation therapy. J Radiat Res.

[31] Chen Y.C., Ke W.C., Chiu H.W. (2014). Risk classification of cancer survival using ANN with gene expression data from multiple laboratories. Comput Biol Med.

[32] Hanai T. (2003). Prognostic models in patients with non-small-cell lung cancer using artificial neural networks in comparison with logistic regression. Cancer Sci.

[33] Hsia T.C. (2003). Prediction of survival in surgical unresectable lung cancer by artificial neural networks including genetic polymorphisms and clinical parameters. J Clin Lab Anal.

[34] Marchevsky A.M. (1998). Artificial neural networks and logistic regression as tools for prediction of survival in patients with stages I and II non-small cell lung cancer. Mod Pathol.

[35] Lander E.S. (2014). Comprehensive molecular profiling of lung adenocarcinoma. Nature.

[36] Hammerman P.S. (2012). Comprehensive genomic characterization of squamous cell lung cancers. Nature.

[37] Quinlan J.R. (1996). Learning decision tree classifiers. ACM Comput Surv.

[38] Grajski K.A. (1986). Classification of EEG spatial patterns with a tree-structured methodology - cart. IEEE Trans Biomed Eng.

[39] Praagman, J., Classification and regression trees - Breiman,l, Friedman,jh, Olshen,ra, Stone, CJ. Eur J Oper Res, 1985. 19(1): p. 144-144.

[40] Schmidhuber J. (2015). Deep learning in neural networks: An overview. Neural Netw.

[41] Auer P., Burgsteiner H., Maass W. (2008). A learning rule for very simple universal approximators consisting of a single layer of perceptrons. Neural Netw.

[42] Hemmat Esfe M. (2015). Applications of feedforward multilayer perceptron artificial neural networks and empirical correlation for prediction of thermal conductivity of Mg(OH)(2)-EG using experimental data. Int Commun Heat Mass Transfer.

[43] Marquardt D.W. (1963). An algorithm for least-squares estimation of nonlinear parameters. J Soc Ind Appl Math.

[44] Cortes C., Vapnik V. (1995). Support-vector networks. Machine Learn.

[45] Suykens J.A.K., Vandewalle J. (1999). Least squares support vector machine classifiers. Neural Process Lett.

[46] Arlot S., Celisse A. (2010). A survey of cross-validation procedures for model selection. Stat Surv.

[47] Chen K. (2019). SNHG7 mediates cisplatin-resistance in non-small cell lung cancer by activating PI3K/AKT pathway. Eur Rev Med Pharmacol Sci.

[48] Hu C. (2019). ROCK1 promotes migration and invasion of nonsmallcell lung cancer cells through the PTEN/PI3K/FAK pathway. Int J Oncol.

[49] Wu Z. (2019). Non-invasive detection of EGFR and TP53 mutations through the combination of plasma, urine and sputum in advanced non-small cell lung cancer. Oncol Lett.

[50] Hung L.V.M. (2019). Nootkatone, an AMPK activator derived from grapefruit, inhibits KRAS downstream pathway and sensitizes non-small-cell lung cancer A549 cells to adriamycin. Phytomedicine.

[51] Lankenau M.A. (2015). MicroRNA-3151 inactivates TP53 in BRAF-mutated human malignancies. Proc Natl Acad Sci USA.

[52] Houston K.A. (2018). Histologic lung cancer incidence rates and trends vary by race/ethnicity and residential county. J Thorac Oncol.

[53] Fairley T.L. (2011). Racial/ethnic disparities and geographic differences in lung cancer incidence-38 states and the district of Columbia, 1998–2006 (Reprinted from MMWR, vol 59, pg 1433–1438, 2010). JAMA-J Am Med Assoc.

[54] Kiyohara C., Ohno Y. (2010). Sex differences in lung cancer susceptibility: a review. Gend Med.

[55] Nakamura H. (2011). Female gender is an independent prognostic factor in non-small-cell lung cancer: a meta-analysis. Ann Thorac Cardiovasc Surg.

[56] Weiss J.M. (2008). Menstrual and reproductive factors in association with lung cancer in female lifetime nonsmokers. Am J Epidemiol.

[57] Schabath M.B. (2004). Hormone replacement therapy and lung cancer risk: a case-control analysis. Clin Cancer Res.

[58] Salminen A., Kaarniranta K., Kauppinen A. (2018). Phytochemicals inhibit the immunosuppressive functions of myeloid-derived suppressor cells (MDSC): Impact on cancer and age-related chronic inflammatory disorders. Int Immunopharmacol.

[59] Detterbeck F.C. (2017). The eighth edition lung cancer stage classification. Chest.

[60] Rami-Porta R. (2014). The IASLC lung cancer staging project: the new database to inform the eighth edition of the TNM classification of lung cancer. J Thorac Oncol.

[61] Perez C.A. (1987). Long-term observations of the patterns of failure in patients with unresectable non-oat cell carcinoma of the lung treated with definitive radiotherapy. Report by the Radiation Therapy Oncology Group. Cancer.

[62] Inal, C., et al., Emerging treatment for advanced lung cancer with EGFR mutation. 2015. 20(4): p. 597-612. 10.1517/14728214.2015.1058778 26153235

[63] Jordan, E.J., et al., Prospective comprehensive molecular characterization of lung adenocarcinomas for efficient patient matching to approved and emerging therapies. 2017. 7(6): p. 596-609. 10.1158/2159-8290.CD-16-1337 PMC5482929 28336552

[64] Mascaux, C., et al., The role of RAS oncogene in survival of patients with lung cancer: a systematic review of the literature with meta-analysis. 2005. 92(1): p. 131. 10.1038/sj.bjc.6602258 PMC2361730 15597105

[65] Johnson, M.L., et al., Association of KRAS and EGFR mutations with survival in patients with advanced lung adenocarcinomas. 2013. 119(2): p. 356-362. 10.1002/cncr.27730 PMC3966555 22810899

[66] 66. Shepherd, F.A., et al., Pooled analysis of the prognostic and predictive effects of KRAS mutation status and KRAS mutation subtype in early-stage resected non–small-cell lung cancer in four trials of adjuvant chemotherapy. 2013. 31(17): p. 2173. 10.1200/JCO.2012.48.1390 PMC4881333 23630215

[67] Macerelli, M., et al., Does KRAS mutational status predict chemoresistance in advanced non-small cell lung cancer (NSCLC)? 2014. 83(3): p. 383-388. 10.1016/j.lungcan.2013.12.013 24439569

[68] Singh, A., et al., A gene expression signature associated with “K-Ras addiction” reveals regulators of EMT and tumor cell survival. 2009. 15(6): p. 489-500. 10.1016/j.ccr.2009.03.022 PMC2743093 19477428

[69] Ferrer, I., et al., KRAS-mutant non-small cell lung cancer: from biology to therapy. 2018. 124: p. 53-64. 10.1016/j.lungcan.2018.07.013 30268480

[70] Tomasini P. (2016). EGFR and KRAS mutations predict the incidence and outcome of brain metastases in non-small cell lung cancer. Int J Mol Sci.

[71] Smit E. (2014). BRAF mutations in non-small-cell lung cancer. J Thorac Oncol.

[72] Lee S.Y. (2015). The influence of TP53 mutations on the prognosis of patients with early stage non-small cell lung cancer may depend on the intratumor heterogeneity of the mutations. Mol Carcinog.

[73] Fawcett T. (2006). An introduction to ROC analysis. Pattern Recogn Lett.

[74] Pollack J.R. (2007). A perspective on DNA microarrays in pathology research and practice. Am J Pathol.

